# 2016: A year of records

**DOI:** 10.1080/20008198.2017.1281533

**Published:** 2017-01-23

**Authors:** Miranda Olff

**Affiliations:** ^a^ European Journal of Psychotraumatology, Department of Psychiatry, Academic Medical Center, University of Amsterdam & Arq Psychotrauma Expert Group, Diemen, The Netherlands

Happy New Year to our readers around the world – some of them it just started, for others it will soon begin – from the *European Journal of Psychotraumatology*, the *European Society for Traumatic Stress Studies (ESTSS)* and the team at *Taylor and Francis*.

According to Johan Norberg (here), a popular Swedish author, historian and expert in economic globalization, our progress has been unprecedented. On almost any index things are markedly better now than they have ever been. We are safer, healthier, there is more prosperity and less inequality.

Yet, we have witnessed a dramatic increase in political, religious and ideological violence around the world. The year 2016 has not been an easy one for many individuals. Terrorist attacks have dominated the news and sadly, the first day of this year did not start well with another act of terrorism in Turkey.

Fear is easily fostered. Although we are much more likely to be in a traffic accident than to find ourselves a victim of terrorism, our mind is not always rational about it. And the minds of those who have witnessed violence in the past are likely to be even more tuned in towards potential danger. A terrorist hidden in a stream of refugees easily feeds beliefs on the dangers of the stranger, making refugees and migrants easy topics dominating the elections all over Europe and in the US.

Man-made or not, global warming has continued: 2016 was the third year in a row with record high global temperatures with floods, earthquakes and draughts affecting millions of people. This also leads to more global inequality and migration. The UNHCR reported that more people have been driven from their homes than at any time since records began, over 65 million displaced worldwide, with the shocking conclusion that ‘one in every 113 people on earth is either an asylum-seeker, internally displaced or a refugee’ (UNHCR, http://www.unhcr.org/global-trends-2015.html). Relatively few asylum seekers find their way to Western Europe, by the way. Countries like Turkey, Pakistan, Lebanon, and Iran host most of them (Turner, 2015). While refugees and migrants are trying to escape from war, draught, poverty, or violence, they represent one of the most vulnerable populations. However, they have the same aspirations as any human being – to be safe, healthy, and with a better future (for their children).

## 1. Trauma professionals can make a difference

Despite these large-scale disasters and violence life goes on in our homes, practices, and communities. Trauma professionals can make a difference, researchers and clinicians, from early responders, to those providing specialized care.

The *European Journal for Psychotraumatology* hit a few records too in 2016: the total number of downloads and citations and its impact factor are at its highest since the launch of the journal, while the time from submission until publication was at its shortest. However, it is, of course, the content that matters and of which I am proud. In 2016 in the *European Journal of Psychotraumatology* we addressed a wide range of issues that matter for patients, practitioners, researchers, and policy makers.

With regard to the refugee crisis we published a special issue *Global mental health: Trauma and adversity among populations in transition* (Hall & Olff, 2016)), we learned for instance that when refugees encounter new traumatic or stressful life events this leads to significant fluctuations in posttraumatic stress, anxiety, and depressive symptoms over the year (Schock, Böttche, Rosner, Wenk-Ansohn, & Knaevelsrud, 2016), and that employment status was important with regard to their treatment outcome (Sonne et al., 2016). With ‘Fresh eyes on the European refugee crisis’ (Alisic & Letschert, 2016) we get insights into the Young Academies’ (selected for the excellence of their research and a firm commitment to translating academic insights into society) views on the refugee problem.

In 2016 we also published other special issues: *Trauma occurs in social contexts (*Sijbrandij & Olff, 2016), the theme of the ESTSS meeting; *Eye Movement Desensitization and Reprocessing therapy research (EMDR) (*Nijdam & Olff, 2016), with EMDR being very popular in Europe it is good to learn about its scientific basis; and *Highlights from ISTSS 2015 (*Elzinga, Schmahl, & Olff, 2016) with many interesting contributions from keynote speakers or featured sessions. I was pleasantly surprised with the openness to new approaches by key leaders in a session on ‘What I have changed my mind about and why’ such as integrative therapies including comprehensive holistic care, exercise, returning to competitive work, logotherapy, mindfulness, and enhancing well-being and resilience (Yehuda et al., 2016).

Please check out our new journal website (www.tandfonline.com/zept) to see what else we published in 2016 and before. With the e-alerts function one can easily receive each newly published paper when it comes out in your mailbox.

## 2. A new publisher

This is the first publication of the *European Journal for Psychotraumatology* with our new publisher. As you may have noticed our previous publisher Co-Action Publishing, pioneers in open access scholarly publishing, has now joined the Taylor & Francis Group. Many thanks to the Co-Action Publishing team for their commitment in making such a good start with our journal, helping us to make a difference in the field of traumatic stress, scientifically, clinically and with societal impact (Olff, 2016).

There are several exciting developments that come with moving to a bigger publishing house such as the potential to reach a larger audience via the new platform as well as marketing and conference activities. Taylor & Francis also offers diverse helpful resources for authors, editors and societies. These include practical elements such as Editorial Manager system to manage peer review, to running author workshops and editorial roundtable events around the globe to support authors and the work of editors, check it out here.

To host the exciting diversity of developments in the field psychotraumatology we now have the following manuscript categories: original basic and clinical research articles that consolidate and expand the theoretical and professional basis of the field of traumatic stress; Review articles including meta-analyses; Short communications presenting new ideas or early-stage promising research; Study protocols that describe proposed or ongoing research; Case reports examining a single individual or event in a real-life context; Clinical practice papers sharing experience from the clinic; PhD thesis Summaries; Letters to the Editor debating articles already published in the Journal; and Book Reviews. Both quantitative and qualitative research is welcome. The *European Journal of Psychotraumatology* is open to innovative, unconventional, or inspirational contributions that move the field forward. All research papers undergo strict peer review.

The fees will still remain relatively low, especially for members of the *European Society for Traumatic Stress Studies (ESTSS)* as ESTSS owns the journal. By the way, all around the world are welcome to join the society.

Some things did not change, I am very pleased to continue with almost the same team of associate editors (Cherie Armour, Chris Brewin, Marylene Cloitre, Anke Ehlers, Julian Ford, Jane Herlihy, Ruth Lanius, Rita Rosner). We have an active editorial board and are very grateful to all board members and external reviewers for their reviewing of papers for the journal.

We will have several guest editors throughout the year for special issues. Ongoing & Planned special issues for 2017 include:

1. *The neurobiology of PTSD (Ruth Lanius)*
2. *Bayesian statistics (Rens van de Schoot)*
3. *PTSD Symptomics: Analyzing individual PTSD symptoms and their network configurations in traumatized populations (Cherie Armour & Eiko Fried)*
4. *ISTSS Highlights 2016 (Paul Frewen & Christian Schmahl),*
5. *Treatment for refugees* (Christine Knaevelsrud) – call soon to come out.
6. *Child Maltreatment Across the Lifespan- highlights of the ESTSS meeting*
7. *Traumatic Grief (Paul Boelen & Geert Smid)*
8. *e-Health for trauma related symptoms*

Finally, we are planning our next Editorial Board Meeting in Denmark at the *European Society for Traumatic Stress Studies* meeting in Odense, Denmark (June 2–5, 2017). Save the date! So we watch the year to come with great interest. I look forward to reading and sharing with you the exciting things that psychotrauma professionals are doing in 2017.

With best wishes for a happy New Year 2017.
